# Strain Monitoring of Concrete Using Carbon Black-Based Smart Coatings

**DOI:** 10.3390/ma17071577

**Published:** 2024-03-29

**Authors:** Gabriele Milone, Christos Vlachakis, Jean-Marc Tulliani, Abir Al-Tabbaa

**Affiliations:** 1 Department of Engineering, University of Cambridge, Trumpington Street, Cambridge CB2 1PZ, UK; cv375@cam.ac.uk (C.V.); aa22@cam.ac.uk (A.A.-T.); 2 Department of Applied Science and Technology, National Interuniversity Consortium of Materials Science and Technology Research Unit, Lince Laboratory, Politecnico di Torino, Corso Duca degli Abruzzi 24, 10129 Turin, Italy; jeanmarc.tulliani@polito.it

**Keywords:** structural health monitoring, self-sensing, carbon black, cement-based materials, smart coatings

## Abstract

Given the challenges we face of an ageing infrastructure and insufficient maintenance, there is a critical shift towards preventive and predictive maintenance in construction. Self-sensing cement-based materials have drawn interest in this sector due to their high monitoring performance and durability compared to electronic sensors. While bulk applications have been well-discussed within this field, several challenges exist in their implementation for practical applications, such as poor workability and high manufacturing costs at larger volumes. This paper discusses the development of smart carbon-based cementitious coatings for strain monitoring of concrete substrates under flexural loading. This work presents a physical, electrical, and electromechanical investigation of sensing coatings with varying carbon black (CB) concentrations along with the geometric optimisation of the sensor design. The optimal strain-sensing performance, 55.5 ± 2.7, was obtained for coatings with 2 wt% of conductive filler, 3 mm thickness, and a gauge length of 60 mm. The results demonstrate the potential of applying smart coatings with carbon black addition for concrete strain monitoring.

## 1. Introduction

The authorities responsible for the existing infrastructure are struggling to keep up with modern safety and performance requirements due to ageing and degradation of the majority of the built environment and lack of regular upkeep. As such, structural assessment as well as continuous and corrective maintenance protocols have become a central part of retrofitting and prolonging the lifespan of existing structures [1]. Current practices include reactive maintenance procedures, which are financially and environmentally taxing [2]. Thus, preventive and predictive maintenance becomes the most plausible direction for the future of the construction sector [3].

Therefore, the exploration of advanced structural health monitoring (SHM) techniques is critical as the construction sector moves towards more sustainable and efficient management strategies. Among various SHM methods, the use of smart cementitious binders, enhanced with electrically conductive fillers, emerges as a promising approach to overcome the limitations of traditional monitoring systems. Specifically, this study focuses on carbon black (CB)-based cementitious coatings, to address the challenges of inadequate data acquisition and structural assessment under loading. Overall, the research aims to contribute to the development of more resilient and efficient infrastructure maintenance protocols, addressing a crucial gap in the current approach to structural health monitoring.

## 2. Background

### 2.1. Advances in Structural Health Monitoring

Structural health monitoring (SHM) aims to manage and evaluate the condition of structures through data acquisition and analysis. Depending on the application, data could be collected through a variety of methods such as fibre optic sensors [4], acoustic emissions [5], computed tomography [6], sensing sheets, [7], satellites [8], and piezoelectric transducers [9]. Despite their widespread use, one of the main limitations of the practical applications of these SHM techniques lies in their performance under aggressive environments, which could be compromised and lead to premature failure and, thus, to inadequate data acquisition and structural assessment [10,11]. In the pursuit of an alternative monitoring method, smart cementitious binders have been investigated over the years for strain and damage sensing [12]. These materials are cement binders, such as pastes, mortar, or concrete, that have been doped with a single or a combination of electrically conductive fillers to enhance their sensing properties [12,13]. Monitoring is made possible through the analysis of electrical changes in resistance under external stimuli such as strain, damage, temperature, and moisture [14]. In cementitious systems, the sensing mechanism is a result of ionic and electronic conduction [12,15,16,17]. Ionic conduction is associated with the movement of ions in the cementitious matrix while electronic conduction is related to the movement of electrons within the filler network [18]. The latter is typically the more prominent form of conduction in cementitious binders as it arises from either the mechanical contact or the tunnelling effect between two adjacent filler particles [15,16]. The sensing performance is quantitatively assessed by the relationship between the fractional change of resistivity and the actual strain in the composite, i.e., gauge factor (GF) [19]. The optimal response for self-sensing cementitious binders has been reported to take place at filler concentrations within the percolation zone [20,21,22]. The percolation zone is defined as the range of filler concentrations in which gains in electrical conductivity can be observed [12,23].

Among the different types of conductive filler, steel and carbon-based materials are commonly used in self-sensing applications [24,25]. The addition of steel-based fillers (e.g., steel fibres, steel wires, and copper-coated steel wires) is generally associated with ultra-high-performance concrete (UHPC) [26]. Recently, some studies have begun investigating the electrical properties of these mechanically enhancing additives for self-sensing purposes [27,28], achieving good electrical conductivity values [29,30] and desirable strain-sensing properties (i.e., gauge factor) [31,32]. Nonetheless, Tang [33] proved that steel fibre concrete is prone to rapid corrosion in the presence of small amounts of NaCl under direct current application. Moreover, in numerous cases, the addition of steel-based additives alone cannot guarantee a sufficient self-sensing performance [30,34]. Therefore, steel fibres are usually coupled with additional conductive fillers to improve the sensing response of the binder [28,35,36], which, in turn, could lead to increased fabrication costs and complexities in mixing. Alternatively, carbon-based materials have been gaining popularity in self-sensing applications due to their high durability [37], good electrical performance [38], and corrosion resistance [39]. Extensive research has been conducted on carbon nanotubes (CNTs) [40,41], carbon nanofibres (CNFs) [42,43,44], natural graphite (NG) [45,46], and graphite nanoplates (GNPs) [47]. Out of the available carbon-based fillers, carbon black (CB) was used in this study as it presents great environmental and financial viability [48,49] due to its production process and the small concentrations required in cementitious binders for electrical enhancement.

### 2.2. Physical and Electrical Influence of Carbon Black in Cementitious Matrices

Carbon black manufacturing processes are divided into thermal–oxidative decomposition and thermal decomposition of hydrocarbons [50]. The former technique exploits the presence of oxygen in open or closed systems (e.g., furnace) while the latter is carried out in closed settings without oxygen and it is sustained by the exothermic reaction of acetylene decomposition [51]. Its main applications are as a pigment or as a thermal/electrical conductive material for the reinforcement of elastomers [52] and plastics [53].

The addition of carbon-based materials in cementitious binders leads to a decrease in free water [54,55], thus affecting the viscosity of the mix. The magnitude of such a change depends on different factors. These variations can be attributed to the different types and quantities of added phases as well as the production protocol pursued [56]. A mix with high viscosity causes poor dispersion of functional fillers, which tend to agglomerate since carbon-based nanomaterials are affected by van der Waals forces between adjacent particles [57]. The formation of agglomerates can compromise the composite’s overall mechanical and electrical properties [58]. Therefore, in cementitious binders, a high-range water-reducing admixture is generally used as a superplasticiser to assist the filler dispersion in the system by increasing the mix fluidity. For what concerns the production of self-sensing materials, carbon black dispersion is generally achieved by first adding the carbon-based filler to a solution of water and superplasticiser [59]. Subsequently, by either mechanical stirring or ultrasonication, the solution achieves sufficient stability to be mixed with cement [58,60,61]. Although sonication is the most effective technique for dispersing nanosized particles, its efficiency is limited by the quantity of solution mixed [62] and, therefore, mechanical stirring has become a more practical solution in this application.

Other than viscosity, this study assessed the influence of carbon black on some of the physical properties of electrically conductive cementitious composites, i.e., flexural strength, hydration products, and rate. In fact, the mechanical strength characterising the nanocarbon-based cementitious binders has been proven to change as a function of the added conductive phase [63]. Furthermore, some works in the literature have specified that the addition of carbon-based materials does not produce new hydration products in cement binders [64]. However, their presence might alter the type, concentration, and growth rate of some of the expected hydration products [65].

Among recent studies, different types [66], concentrations [62,67,68], and combinations [61,69] of carbon black have been applied and investigated in cement binders to achieve electrical enhancement. The electrical conductivity in cementitious systems has been reported to reach a maximum value of 22.0 S/m for 10.0 wt% CB [67]. However, the electrical performance of carbon black in cement does not depend exclusively on the filler concentration but also on its size and carbon purity. To provide an example, by adding 2 wt% of CB, Pisello et al. [70] achieved a conductivity of 1.3 × 10^−5^ S/m for an average particle size of 30 nm, while Hussain et al. [36] reached 4 × 10^−2^ S/m for the same CB concentration with an average particle size of 40 nm. Such a significant variation can also be related to the mix composition. Analogously, Qasim et al. [71] tested three different grades of commercial carbon black in mortars and obtained a conductivity that ranged between 1.2 × 10^−3^ S/m and 2.5 × 10^−2^ S/m for the same filler dosage. Throughout the literature, different combinations of carbon black in cement paste and mortar have been used to achieve a sufficient strain monitoring performance, as summarised in Table 1. A general trend can be observed from past works where cement paste composites resulted in a less conductive but more responsive system compared to mortar and concrete binders [72], i.e., by adding 3 wt% of CB, Baeza et al. [73] obtained mortar samples with a gauge factor of 12.9 while Dong et al. [60] reported a value of 330 for cement pastes with the same CB dosage. This behaviour was attributed to the presence of additional aggregates, which were assumed to improve the conductivity by densifying the electrical network [74,75]. At the same time, the contribution of aggregates in mortar and concrete increased the elastic modulus and reduced the inner displacements of the different components when subjected to load and, therefore, influenced the overall strain-sensing ability [72]. Thus, the work here presented focused on the electrical properties of cement paste with the addition of carbon black.

### 2.3. Electromechanical Sensing Property of Smart Cementitious Sensors

#### 2.3.1. Bulk Applications

The electrical enhancement driven by adding these carbon black-based materials can be exploited for a series of purposes: temperature [76,77], corrosion [55], hydration [78], and humidity sensing [79,80]. When it comes to the strain-sensing capability, Monteiro et al. [67] tested mortar cubes under a cyclic compressive load by applying 12 V in DC. Samples with 7 wt% of added CB resulted in a gauge factor of ~30, while specimens with a CB content of 10 wt% were characterised by a weaker strength and a lower strain sensitivity value of 24. Pisello et al. [70] achieved a gauge factor of 340 for paste samples under cyclic compressive loading with a CB addition of 2 wt%. D’Alessandro et al. [62] obtained a sensitivity value of 169 for 1.5 wt% and 47 for 2 wt% of carbon black-based samples under monotonic compression loading. Similarly, in the study by Hussain et al. [36], they defined a gauge factor of 134 when comparing a lower nanocarbon black addition (1.5 wt%) to a higher dosage (2 wt%), which resulted in a reduced sensitivity value of 96. Zhang et al. [81] investigated the effect of a decreasing water content for cement-based materials with 2.14 vol% of added CNT/CB and found a gauge factor decrement from 389 to 202. Dong et al. [82,83,84] obtained a good electrical relationship with the applied compressive stress for cementitious systems both with microencapsulation of carbon black enclosing slaked lime for self-healing purposes and with added polypropylene (PP) fibres for structural enhancement. In a continuation to cyclic compressive loading, Lima et al. [85] did not obtain any piezoresistive response from mortar samples with CB additions below 3 wt%, while achieving a maximum average gauge factor of 516 for 6 wt% of carbon black. Similarly, Nalon et al. [86,87] observed peak strain sensitivity (GF~1000) in 9 wt% CB-based mortars rehydrated after 200 °C exposure. They also found that at the end of the percolation zone, a reduction in the contact mechanism led to an increment of strain sensitivity, i.e., from a gauge factor of 154 to 205 by adding lime [88] and from 255 to 437 due to the presence of expansive agents [89]. It should be pointed out that all the aforementioned research, displayed in Table 1, focused only on compressive strain sensitivity. In fact, fewer studies have investigated smart cementitious composites for alternative types of loading. In this regard, Li and Li [69] investigated the sensing performance of CB-based mortars for both tensile and compressive cyclic loading. When applying an alternating current (AC) with a frequency of 5 MHz, they found tensile gauge factors of 52, 247, and 105 for filler dosages of 2.5, 5, and 10 vol%, respectively, with a similar pattern for the samples in compression, i.e., gauge factors of 81, 344, and 236, with respect to the same filler concentrations. This sensitivity variation between tension and compression was also described by Han et al. [90]. In fact, under cyclic loading, the authors obtained a compressive gauge factor of ~166 and a tensile factor of ~15 for the same CB addition (i.e., 0.6 vol%). Such a sensing response variation has been attributed to the filler’s influence on the micromechanical parameters of the composite under different load conditions [69]. In tension, carbon black can further impact crack propagation [91] and becomes progressively separated through crack formation, thus reducing its contribution to the conductive pathway. In contrast, a compressed matrix causes the convergence of initially distant particles and, thus, a more continuous electric network [90].

**Table 1:** Previous research on self-sensing cementitious composites with carbon black additions. All samples were produced by mechanical stirring/ultrasonicating a solution of CB/water/SP before adding cement powder. The strain-sensing performance was obtained by comparing the electrical response of the system with its compressive strain, measured with commercial strain gauges or clip gauges. Coefficient of correlation and repeatability were used to evaluate the accuracy of the results given. For comparison purposes, the values shown for [86,87] have been selected from measurements at 25 °C.

Reference	Cementitious Type	CB Dosage [wt%]	Electrical Conductivity [S/m]	Gauge Factor
[70]	Paste	2.0	1.3 × 10^−4^	340
[21]	Paste	1.0	1 × 10^−4^	96
[62]	Paste	1.5	0.9 × 10^−4^	169
		2.0	40 × 10^−4^	47
[66]	Mortar	8.0	1.2	95
[67]	Mortar	7.0	0.47	30
		10.0	22.0	24
[48]	Mortar	1.5	0.22	57
[85]	Mortar	5	5.88 × 10^−2^	111
		6	38.5 × 10^−2^	516
[86]	Mortar	6	0.56 × 10^−2^	150
		9	111.1 × 10^−2^	375
[36]	Concrete	0.3	4.8 × 10^−3^	11
		0.5	5.7 × 10^−3^	15
		0.8	7.7 × 10^−3^	34
		1.0	8.0 × 10^−3^	57
		1.3	8.3 × 10^−3^	110
		1.5	20 × 10^−3^	134
		1.8	20 × 10^−3^	110
		2.0	40 × 10^−3^	96
[71]	Mortar	10	2.49 × 10^−2^	224
		12.5	2.76 × 10^−2^	141
		15	3.87 × 10^−2^	185
[87]	Mortar	6	0.56 × 10^−2^	390
		9	111.1 × 10^−2^	530
[88]	Mortar	6	0.11	253
		9	0.67	154

Although the strain-sensing performance of carbon black-based composites is well investigated in compression and is expanding in tension, its behaviour under flexural strain is still at an earlier research stage. Among the few available studies, Guo et al. [92] investigated the self-sensing capability of a hybrid system composed of carbon black and polypropylene fibres under a cyclic bending load. They obtained a linear relationship between the applied stress and the electric response for 0.5 wt% of PP fibres and 1.5 wt% of added CB. Analogously, Dong et al. [83] investigated the correlation between the electric performance and the applied monotonic flexural stress for prisms with hybrid PP/CB additions. This stress/electrical relationship peaked at 0.55%/MPa for cement pastes with 0.5 wt% carbon black and 0.4 wt% PP fibres; without the polymeric fibres, the maximum stress sensitivity achieved was 0.06%/MPa [83]. Alternatively, Ding et al. [34] tested concrete with 0.25 wt% of added carbon black and varying concentrations of steel fibres (i.e., 5, 10, and 15 wt%). Depending on the steel fibre dosage, a linear or bi-linear relationship was found between the electrical response and the crack opening displacement that ranged from 1.89 to 4.68%/mm.

#### 2.3.2. Coating Applications

When it pertains to strain monitoring, self-sensing cementitious binders are typically fabricated either as full-sized construction materials—defined as the “bulk form” [21,62,66,67,70]—or as small binders that are embedded in larger structures [58,93,94]. However, self-sensing cementitious materials can also be applied to existing infrastructure for sensing purposes in the form of coatings [95]. Coatings provide greater flexibility in terms of deployment for new and existing infrastructure, and also in fabrication due to their smaller size and thus lower costs [14,93]. When compared to traditional electronic sensors employed in the construction field, e.g., strain gauges, sensing coatings offer the advantage of monitoring broader areas instead of individual points within structures. While alternative sensors, such as fibre optics, could meet this criterion, they often necessitate expensive analysers for operation and are prone to fibre breakage during installation [96]. In general, conventional sensors come with disadvantages such as limited durability, low sensitivity, and poor compatibility with concrete structures [94]. Cement-based sensing coatings, on the other hand, boast a simpler installation process and can operate with cost-effective electrical devices. Moreover, these coatings achieve a greater interaction with the substrate as well as higher flexibility in monitoring as they are capable of pivoting from strain-sensing [97] to damage-sensing and tomography applications [98]. Lastly, beyond their sensing capabilities, cementitious coatings can also serve as an effective retrofitting solution for structural or non-structural repairs [99].

Despite their distinct advantages, however, the investigation of coatings in the literature is rather limited when compared with self-sensing structural elements [66,90,100]. The concept of sensing coatings was pioneered by Wen and Chung [101], in which carbon fibres were added to cement paste to monitor the tensile and compressive strain in cementitious beams under flexural loading. The resistance of the coating in the tensile region increased upon flexure (i.e., 0.15%) while the coating in the compressive region decreased (i.e., 0.06%). Baeza et al. [102] investigated the compressive and tensile strain-sensing response of carbon fibre coatings attached to reinforced concrete (RC) beams under bending. The authors reported compressive gauge factors of 176 and 192 for different coating sizes when cast in situ onto an RC beam. In the tensile region, the gauge factors reported were between 178.9 and 64.8 for carbon-fibre-based coatings with varying thicknesses. It was conjectured that greater strain transmission occurred in thinner coatings, thus leading to a greater gauge factor. Likewise, Durairaj et al. [103] applied carbon fibre and brass fibre mortar coatings to concrete beams under flexural bending for damage detection while employing different coating attachment methods; although a dependency between the electrical properties and the applied load was evident, the strain-sensing sensitivity was not calculated. Moreover, Kim et al. [104] investigated the sensing behaviour of multi-walled carbon nanotube (MWCNT)–cement repairs under bending, and while a change in electrical properties under a load was observed, the sensing coefficient of the repairs was not determined. Despite the financial and safety benefits of carbon black over existing conductive fillers, the use of CB in sensing coatings is rather scarce. Existing studies have focused primarily on compressive strain sensing in which the GF ranged between 123 and 490 [90]. A recent work by Qiu et al. [105] described the sensing response in specific areas of mortar beams with mixed inclusions of carbon black and carbon nanotubes. They obtained a maximum FCR within the elastic flexural loading of 1.5% in compression and 2.5% in tension, for a filler addition of 2 vol%. The mortar’s response significantly increased to 55.9% and 31.3% when reaching damage conditions.

### 2.4. Research Significance

Overall, while studies on self-sensing coatings exist, comprehensive investigations that include workability, adhesion [106], and proper strain characterisation on self-sensing CB coatings are rather limited. To remeday that, this paper aims at the holistic investigation of the use of carbon black-based smart coatings for flexural strain monitoring. Furthermore, this study introduces an experimental protocol—from manufacturing to application—to be used as a starting point for the development of such coatings and their employment in the field of structural health monitoring. This paper initially assesses the effects of CB through the electrical and physical characterisation of cement pastes. The produced sensing coatings are then applied onto concrete substrates and their strain-sensing response is investigated under cyclic flexural loading. Finally, the sensing coating design is optimised in terms of coating thickness, electrode spacing, and CB dosage to achieve the highest sensing performance.

## 3. Materials and Methods

### 3.1. Materials

Smart coatings were produced by combining Portland cement (CEM I—52.5N), supplied by Heidelberg Materials, Maidenhead, UK and conforming to BS EN 197-1 [107] (ASTM equivalent: C150 [108]), with conductive carbon black powder, supplied by Alfa Aesar, Ward Hill, MA, USA. The characteristics of the CB are shown in Table 2.

The concrete substrate was produced by adding sand and coarse aggregate to the cementitious mix (BS EN 206-1, ASTM C33/C33M-23 [109,110]). The sand was characterised by a maximum size of 2 mm and a specific gravity of 2.56 and water absorption of 0.6%, while the maximum size of the coarse aggregate was 11.2 mm with a specific gravity of 2.58 and water absorption of 1.8%, similarly to that used and characterised in previous work [111]. Table 3 shows the mix design used for casting concrete beams for electromechanical testing, which resulted in a high-density mix (2595 kg/m^3^) to facilitate sample preparation, compaction [112], and subsequent coating application.

When it pertains to electrode selection, even though the use of steel meshes as electrodes leads to a uniform and reliable electrical analysis within the conductive matrix [31,58], such practice excessively weakens the designed coating structure. Therefore, in view of a more feasible application, 20 mm long and 1 mm thick copper wires—supplied by RS Components, Corby, UK—were embedded across the coating to achieve good electrical continuity throughout the system [19].

### 3.2. Sample Preparation

**Figure 1:**
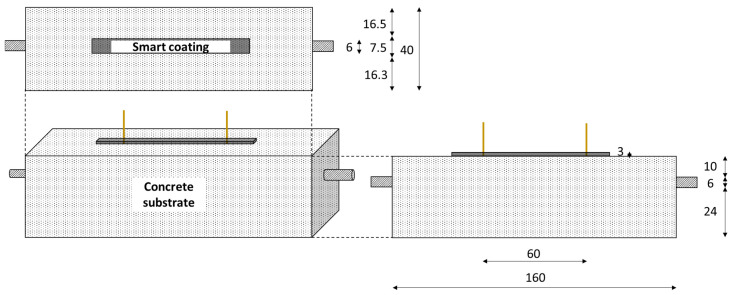
Schematic representation of the substrate/coating system produced for testing. The image shows the smart coating with 3 mm thicknesses and 60 mm electrode placement for the 2-probe method (all dimensions in mm). The beam/coating system has been reversed in the image for better clarity. The coating is here shown with some transparency to better present the embedment of the copper wires used as electrodes.

This study focused on the strain monitoring of concrete beams (40 mm × 40 mm × 160 mm) with the use of electrically conductive CB–cement paste coatings (7.5 mm × 3 mm × 80 mm); the coatings were applied to the region of the beam that was subjected to tension—as shown in Figure 1. It is crucial to specify that concrete was chosen as the substrate material in view of real scale applications, conforming with the basic dimensions for the maximum diameter (BS EN 12390-1 [113]). Nonetheless, given the laboratory scale, the beams were adequately compacted and visually assessed for this application. Moreover, the substrate’s dimensions were chosen in agreement with similar works on flexural strain-sensing systems [101,104], while the coating size was tailored to the substrate in view of large-scale applications. The concrete beams were produced following a weight ratio of 1:2.1:3 of cement:sand:coarse aggregate. To improve the concrete response to the applied cycles of flexural load, a 200 mm long rebar with a diameter of 6 mm was embedded along the portion of the substrate subjected to tensile stress, i.e., in Figure 1, the bar is 10 mm below the top layer—which represents the tensile section—to support the mechanical performance of the beam under tensile stress. The rebar diameter was selected according to the minimum longitudinal reinforcement of concrete set by the standards (BS EN 1992-1 [114]).

Figure 1 also shows the substrate/coating layout for a 3 mm thick coating and 60 mm electrode spacing. In Section 4.4, we outline how different coating configurations were tested with a thickness of 9 mm and an electrode distance of 10 mm. The electrode locations were chosen to represent the whole constant bending moment region (60 mm) and the closest possible configuration (10 mm). The electrodes were vertically embedded in the coatings during casting. Their application was aided by the use of small pincers that controlled the position of the electrodes and prevented movement during casting and curing. To ensure that the thickness of the sensors was in line with their nominal value, five thickness measurements were obtained for all coatings along their longitudinal direction by means of a calliper. The resistivity was calculated on this basis to prevent the sensitivity aim of this study from being influenced by any geometrical variability.

**Figure 2:**
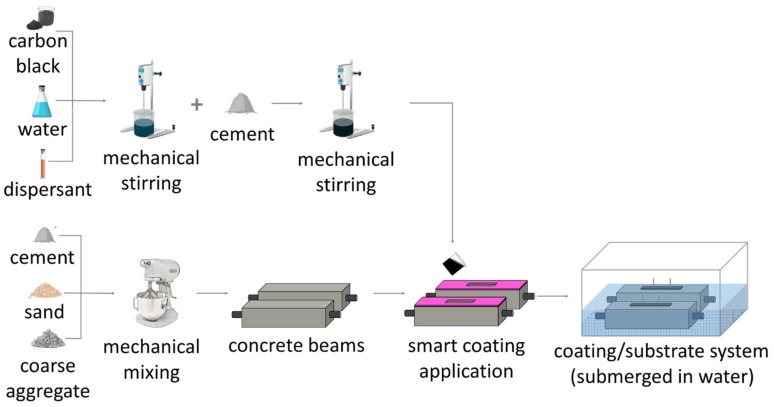
Schematic representation of the mixing steps of CB–cement paste coatings and concrete substrates. First, concrete is mixed and cast in 40 mm × 40 mm × 160 mm moulds. After 3 h, the conductive CB/cement mixture is produced and applied on top of the concrete substrate in 7.5 mm × 3 mm × 80 mm silicon moulds. Finally, the composite system is left to cure in water for 28 days.

As shown in Table 4, the mixtures were fabricated with a water-to-cement ratio of 0.45 and varying CB contents of 0.1, 1.0, 2.0, 2.5, 3.0, 4.0, and 5.0% by the weight of cement. The studied carbon black concentrations were chosen according to the section of the percolation curve that showed for analogous works an abrupt variation in the composite’s electrical performance for small filler variations [48,66]. A few studies investigated the influence of higher carbon black dosages, i.e., 8 wt% [115], 10 wt% [67], and 15 wt% [116]. This work, however, tested a maximum CB addition of 5 wt% due to the challenges that higher dosages of carbon black impose on the workability conditions. While producing the CB-based mix, filler concentrations of 5 wt% led to a mix viscosity of ~1 × 10^3^ mPa∙s, precluding, therefore, any further carbon black casting and mixing. In fact, regardless of the added dispersant, the use of high quantities of conductive filler causes a viscosity increment proportional to the filler dosage related to the free water reduction [54,55,64]. For the production of the sensing coating, firstly, a solution of water and dispersant was mixed with the conductive powder. Different percentages of MasterGlenium C315—supplied by BASF, Stockport, UK—were applied by weight of carbon black, providing consistent workability for the varying CB concentrations investigated. Then, the CB-based solution was subjected to mechanical stirring by an *IKA rw20*, UK, mixing probe at 4000 rpm for 6 min. The cement was added to the homogenous solution and mechanically stirred for 5 min at 5000 rpm until the mix became sufficiently homogenous to be cast in silicon moulds, which were placed on top of the concrete beams (whose curing time reached 3 h), as shown in Figure 2. Subsequently, the two copper wires were embedded in the coating, ensuring they did not penetrate the concrete substrate.

The substrates were first cast and left to cure for three hours, to exceed their initial setting time, before applying the sensing coating. This time gap was chosen to allow the substrate to set to a certain extent such that the sensing coating would not submerge nor mix with the substrate. At the same time, to ensure the proper adhesion of the coating with the substrate and avoid the use of bonding agents, this delayed application should not be protracted excessively. Thus, after preliminary tests, a delay of three hours proved to be effective in reaching these goals. Once the coating was applied to the substrate, the entire structure was then wrapped in plastic film and demoulded after 24 h and subsequently submerged in water for 28 days, in agreement with BS EN 13670 [117] and BS 8500 [118] (ACI equivalent: 308R and 301 [119,120]). After curing, the beams with the attached coatings were placed in a high-humidity chamber—maintaining a stable saturation degree of 90%—until electrical testing. Figure 2 schematically shows the mixing and fabrication procedure of the smart coatings and their application on concrete substrates.

### 3.3. Experimental Program

#### 3.3.1. Physical Testing

Firstly, thermogravimetric analysis (TGA) (PerkinElmer, Buckinghamshire, UK) was pursued to estimate the mass loss of volatile components in CB powder, to measure the material’s thermal stability and to ensure its purity. The initial mass of carbon black was about 20 mg. The TGA experiment was performed in air, the temperature ranged from 150 to 900 °C, at a heating rate of 10 °C/min, and the gas flow rate was kept constant at 30 mL/min.

As the fluidity characteristics of the cementitious systems varied as a function of the added concentration of carbon black and superplasticiser content, the rheology of the conductive pastes was assessed. The testing protocol was carried out at room temperature through a smooth-walled DV3T rheometer—supplied by AMETEK Brookfield, Harlow, UK—with an SC4-27 spindle. It consisted of five minutes of rest for stabilisation followed by seven ascending and six descending equal-rate intervals. The Bingham mathematical model was used [121] to define the composites’ plastic viscosity by linearly fitting the final seven intervals from the obtained shear stress vs. shear rate curves. The shearing profiles were chosen in agreement with the typical casting process [122] and the obtained values had a margin of error defined by testing each mix three times.

Next, this study assessed carbon black’s physical and electrical influence on cement paste. Via an isothermal calorimeter I-CAL 2000 HPC—Calmetrix, Maharashtra, IN, USA—the exothermic reactions of the composites were correlated to the presence of the conductive phase. The test lasted 45 h and involved a total weight of 80 g for each of the following samples: control cement paste, paste with 3 wt% CB addition, and paste with 3 wt% CB addition and 10% dispersant by weight of CB. The test focused on assessing the hydration growth of a cementitious paste with carbon black and the influence that superplasticiser had on its development. Consequently, a single filler dosage—3% by weight of cement—was selected for scrutiny as it represented the most significant concentration for cementitious binders in the context of electromechanical tests, as shown in Section 4.4.

**Figure 3:**
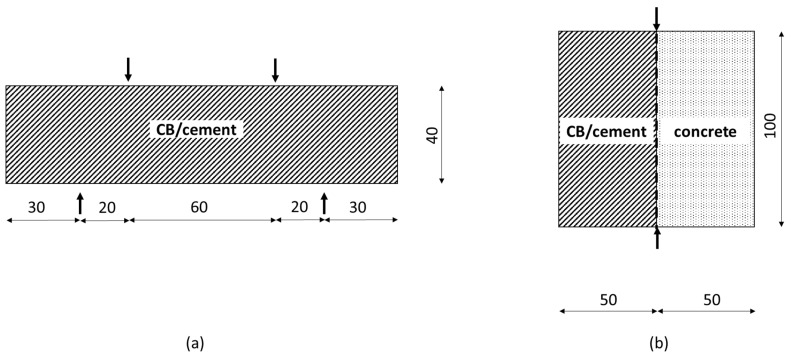
Schematic representation of setup for (**a**) 4-point bending test for flexural strength of CB/cement beams and (**b**) splitting tensile test for CB/cement and concrete adhesion strength (all dimensions in mm).

The application of a flexural load for strength and sensing tests was accomplished with an Advantest9 Uniframe machine—supplied by CONTROLS, Milan, Italy. A 4-point bending test, with a loading rate of 50 N/s, was used to determine the flexural strength of 40 mm × 40 mm × 160 mm prisms of CB–cement paste (shown in Figure 3a) according to Equation (1):

(1) $$f_{b}=\frac{3}{4}\frac{PL}{bd^{2}}$$

where *f_b_* [MPa] is the flexural strength, *P* [N] is the maximum load at failure, *L* [mm] is the support span, *b* [mm] is the width of the test beam, and *d* [mm] is the depth of the beam. The flexural strength was obtained by testing three identical samples for each carbon black dosage to provide a certain redundancy to the measurement.

Given the low tensile behaviour of the concrete layer, whose failure would have posed a challenge in determining the true adhesion strength with the coating, splitting tensile bond tests were conducted as the most suitable option to assess the adhesion strength between the concrete substrate and the smart coating [123,124,125]. A 100 mm × 100 mm × 50 mm CB–cement paste prism was cast on top of a 3 h old concrete element of the same size (Figure 3b). This 3 h delayed application of CB–cement, which was also followed for the electromechanical tests, was chosen to provide sufficient hardening to the substrate while achieving good bonding with the conductive coating. By applying a load rate of 500 N/s, the bond strength was obtained as shown in Equation (2):

(2) $$f_{ct}=\frac{2F}{\pi Ld}$$

where *f_ct_* [MPa] is the bond strength, *F* [N] is the load at failure, *L* [mm] is the load length, and *d* [mm] is the height of the tested element, in agreement with BS EN 1992-1-1 and BS EN 12390-6 [126] (ACI 318 [127] and ASTM C496 [128]). Similar to the bending test, the bond strength was defined by testing three identical cubic samples.

#### 3.3.2. Electrical Testing

Unlike the 4-probe method, the electric current and the voltage drop were measured via two probes, assuming an ohmic behaviour for the composite. This technique tends to be affected by contact resistance whose magnitude is assumed to be lower than the conductive specimen itself [129]. Thus the 2-probe method is capable of measuring changes in electrical properties [130] and provides a simpler setup compared to the 4-probe method [131], limiting the potential for a high stress concentration and potential fractures within the matrix. Piro et al. [132] discovered that the resistivity measurements from the 2- and 4-probe methods are correlated through a constant value, indicating the probe’s impact on resistance. This finding suggests that external factors like contact resistance uniformly influenced all measurements in this study, where identical samples exhibited similar electrical characteristics. The electrical parameters shown in Section 4.2 and Section 4.3 were obtained by electrically testing samples the same size as coatings that were not applied onto concrete substrates. This was carried out to avoid interference in the measurements from the substrate and focus solely on the electrical properties of the CB coatings.

Ohm’s law determines the equivalent electrical resistance between the probes by considering an ohmic behaviour for the composite. As D’Alessandro et al. [40] suggested for DC measurements, the voltage was applied for 10 min to limit the polarisation effect during measurements. The conductivity values were obtained according to Equation (3):

(3) $$\frac{I}{U}\frac{L}{A}=\sigma_{DC}$$

where *I* is the intensity current [A], *U* is the obtained potential drop [V], *L* is the distance between the electrodes or gauge length [mm], and *A* is the cross-sectional area of the part of the electrode that is in contact with the binder [mm^2^].

Additionally, the application of alternate current was implemented using a potentiostat PGSTAT204 (Metrohm, Herisau, Switzerland) to minimise the polarisation effect in the system. The frequency interval of 20 Hz–300 kHz and the amplitude of 0.5 V were chosen according to the literature [46] to measure 9 points per decade. The obtained Nyquist plot is composed of the imaginary–reactance Z″(f) and real–resistance Z′(f) part of the impedance as a function of the frequency value [133,134].

**Figure 4:**
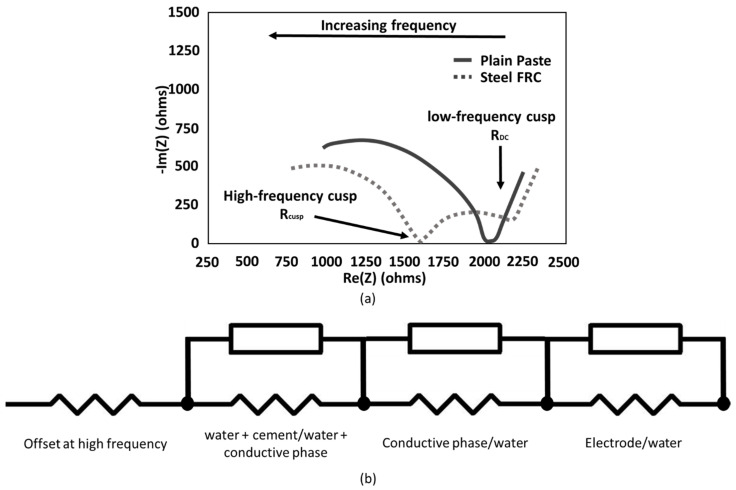
(**a**) Nyquist plot with highlighted cusp points for steel-fibre-reinforced concrete (adapted from [135]) and (**b**) capacitive/resistive components in the equivalent circuit of cement-based conductive composites (adapted from [138]).

Ozyurt et al. [135] and Ferrara et al. [136] defined the comparable value of AC with DC as the cusp point (Figure 4a), i.e., the impedance value at which the reactance is the lowest and its response is almost purely resistive. By interpreting and deconvoluting the obtained impedance spectrum in its equivalent circuit (Figure 4b), the bulk resistance can be used to define the effective conductivity, which is defined by Equation (4) [137]:

(4) $$\frac{1}{R_{bulk}}\;\;\left(\frac{L}{A}\right)=\sigma_{bulk}$$

where *R_bulk_* is the resistance value corresponding to the ionic conduction of the interconnected pores in parallel with the electronic conduction through the conductive filler [138] [Ω], *L* is the distance between the pair of chosen electrodes or gauge length [m], and *A* is the cross-section of the specimen [m^2^]. Moreover, selecting the cusp point for AC applications limits the moisture influence and thus the contribution of ionic conduction in the electrical response of the coatings. To elaborate, Chung [139] specified that the dependency on ionic conduction for humid systems is particularly high and it can strongly change by varying external conditions (e.g., humidity, temperature). Therefore, the capacitance contribution to the AC electrical response—represented by the imaginary part of the impedance—was limited to its minimum by choosing the frequency of the cusp point and, thus, making the measured resistance less dependent on any moisture variation. In this study, electrical tests on smart cement were conducted with the aim of maintaining the system’s inner moisture at a constant 90% saturation degree, a condition identified in analogous studies as optimal for enhancing low-resistance and high strain-sensing capabilities [138,140]. This specific moisture level not only aligns with best practices for smart cement production but also addresses the challenge of water theft, when water is drawn into the concrete substrate from the coating after application and full curing. By maintaining a high saturation degree, this phenomenon was effectively limited, ensuring that the sensors retained their electrical and physical properties throughout the testing period.

#### 3.3.3. Electromechanical Testing

Once the electrical characteristics were obtained, the strain-sensing properties of the coatings were analysed by investigating their changes in resistivity under applied load, i.e., fractional change in the electrical resistivity (FCR) [58]. The electromechanical tests involved only AC measurements to assess the coating’s electrical response to flexural load due to its higher reliability and equipment precision. Due to the electrode’s configuration and the coating/substrate interface, the current lines might propagate to the substrate, affecting the coating’s electrical response. Nonetheless, as the electrode position was ensured to be the same for all tested samples and the electromechanical tests did not focus on the absolute resistivity value but rather on the fractional change of resistivity, the comparison between all samples was possible as they were equally affected by the same setup. The tested prisms were subjected to 20 cycles between 0.84 MPa (0.6 kN) and 4 MPa (2.84 kN), with a loading rate of 50 N/s, in a standard laboratory testing environment. The load interval was chosen to be below 20% of the ultimate flexural strength of the concrete prism to ensure the elastic strain response of the system under bending. The lower threshold—5% of the beams’ flexural strength—was selected as the minimum preload that was required by the coating to start monitoring the cyclic strain behaviour. The strain value (ε) at the midspan of the concrete beam was obtained through digital image correlation (DIC). The surface of each sample was coated with a white layer while black points were randomly sprayed for displacement tracking. The obtained images were processed via GeoPIV-RG [141]. When the material is subjected to an external load, the relationship between FCR and the induced strain is defined as the piezoresistive effect [142]. The closed expression in Equation (5) characterising the relationship between the strain and the electrical property of the system is derived from the analogy between the 2D elastostatic field under antiplane shear loading and the 2D electrostatic field [143,144].

(5) $$FCR=\frac{\rho -\rho_{0}}{\rho_{0}}=\lambda \cdot \varepsilon$$

where *ρ* is the instantaneous resistivity, which is a function of the state of strain and the conductive filler, *ρ*_0_ is the initial resistivity value (i.e., when no load is applied), which depends mainly on the conductive filler, *ε* is the strain, and *λ* is the angular coefficient of the *FCR-ε* curve. Finally, the strain sensitivity was obtained by the slope of the linear fit for the FCR vs. strain curve, defined as the fractional change in resistance per unit strain or gauge factor (GF).

It is worth mentioning that a preliminary study from the authors also investigated the strain sensitivity of control samples, i.e., when no carbon black was included in the mix. It was found that, although characterised by some electrical conductivity value, their strain-sensing response was inconsistent when compared to CB-based cementitious composites (similar to what described in [66]). This performance was attributed to the conduction mechanism in plain cement pastes, which is ionic-driven only and does not rely on any additional functional filler in sensing the state of strain within the matrix [12,58]. Moreover, as the effect of the electrodes was not decoupled from the ionic conduction, sensing measurements of control samples were not functional in assessing the influence of the electrodes. In conclusion, this work does not present a strain-sensing test of control samples; instead, it compares the monitoring response of smart coatings with different filler additions, assuming that all measurements contained a consistent and equal electrode resistance error.

## 4. Results

### 4.1. Carbon Black Characterisation

#### Thermogravimetric Analysis

**Figure 5:**
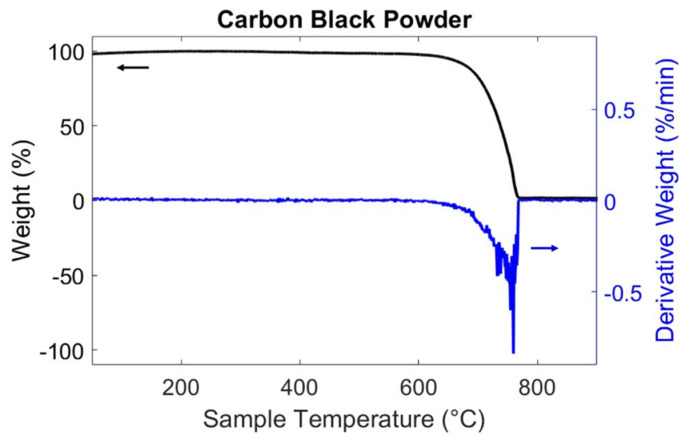
Weight and derivative weight for CB powder. Powder weight stable up to 600 °C. After that temperature, carbon black starts evaporating, to completely dissolve at 765 °C.

Figure 5 displays the thermogravimetric analysis that was conducted to measure the CB’s thermal stability and, therefore, its carbon purity. This result is important, as some organic compounds can strongly influence hydration reactions and, in some cases, even inhibit them [145]. The weight loss for the CB is presented in Figure 5, where the powder is stable until reaching 600 °C, after which it starts oxidating, forming carbon oxides [146]. Its decomposition was continuous up to 765 °C, similar to what was found by Nalon et al. [86,146]. The test evidenced that, due to its high production temperature, carbon black particles did not present any remaining hydrocarbon [112], thus proving the carbon purity of the powder and its suitability for the electrical enhancement objective of this work. Based on this preliminary observation, we propose that future studies should focus on the effect of carbon black when mixed in varying dosages to the cementitious matrix, assessing the composite’s thermal stability [86,146,147].

### 4.2. Influence of CB Dosage on the Physical Properties of the Coating Composite

#### 4.2.1. Viscosity

**Figure 6:**
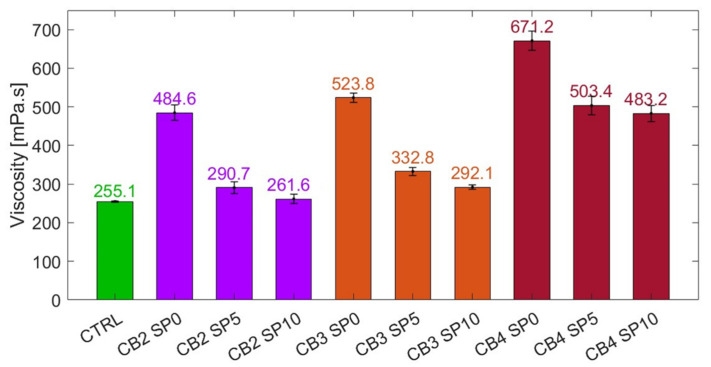
Viscosity measurements for control, CB2, CB3, and CB4 with 0, 5, and 10% dispersant by weight of carbon black. Standard deviation was obtained by testing any specific mix three times.

Since the rheological characteristics of the fresh mix have been found to affect the dispersion of the carbon particles [135] and the overall sensor response evaluation [148], the mix viscosity was assessed and is introduced in this section. The graph in Figure 6 illustrates the viscosity change when adding carbon black. The choice to test CB2, CB3, and CB4 mixes was driven by the electrical tests previously introduced and the strain-sensitivity tests discussed below. In fact, for a shear rate interval of between 8 and 60 s^−1^—consistent with casting protocols—adding 2, 3, and 4 wt% CB led to lower workability, reaching viscosity increments of 90, 105, and 163% (484.6, 523.8, and 671.2 mPa∙s), respectively, compared to the control specimen. The viscosity threshold for casting purposes was empirically found to be around 450 mPa∙s.

It is also evident in Figure 6 that the use of a dispersant was able to reduce the composite’s viscosity. However, excessive viscosity reduction can lead to a poor sensing response [149]. Thus, when adding 10% by weight of the superplasticiser, the systems experienced viscosity decrements of 46, 44, and 28% for CB2, CB3, and CB4, respectively, when compared to the same mixes without dispersant. Therefore, for small carbon black concentrations—below 3 wt%—the addition of 10% of dispersant by weight of CB led to fluidity parameters similar to cement paste (i.e., 261.6 mPa∙s for CB2 and 292.1 mPa∙s for CB3). Such a dispersant amount, although necessary to increase the mix fluidity, was limited in its efficacy by large carbon black concentrations due to the conductive particle’s large surface area. It could be posited that the water in the composite was insufficient for the superplasticiser to facilitate CB dispersion and considerably improve the overall workability [59]. Thus, future work shall focus on further optimising the mix fluidity for high carbon black dosages.

#### 4.2.2. Hydration Growth

**Figure 7:**
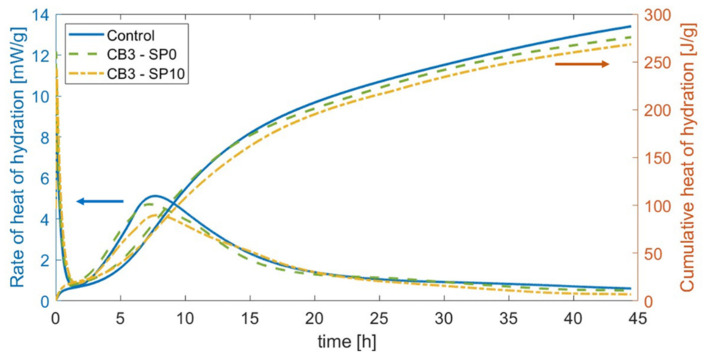
Rate of heat of hydration and cumulative heat of hydration for CTRL, CB3, and CB3 with 10% by weight of CB of dispersant.

The following section presents the influence of carbon black on the fresh properties of cement matrices. Figure 7 indicates that cementitious mixes with CB additions at 3.0 wt% resulted in slightly faster heat production compared to cement pastes during calorimetry testing. Although characterised by a lower rate of heat of hydration, the initial setting time occurred ~30 min earlier than the 4 h needed by cement paste after its casting. Moreover, Figure 7 shows that the addition of superplasticiser slightly delayed the early hydration of the composite, reaching similar timelines to control samples and a slightly slower progression for the heat of hydration.

**Figure 8:**
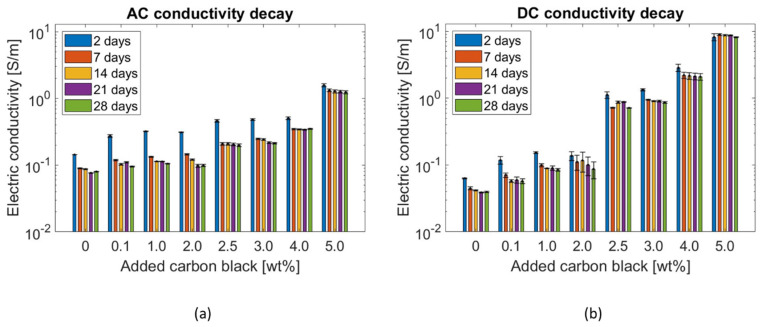
Conductivity decrement for CB/cement specimens within the first 28 days of curing for (**a**) AC and (**b**) DC. All samples experienced a conductivity decay that reached a plateau after 28 days ~25% smaller than the initial conductivity value at 2 days of age. Refer to online version for colour representation.

As the electrical properties of the composite can be linked to the dynamics of the pore solution [78], an additional electrical measurement was conducted that characterised the water present in the composite. Through electrical testing, the exponential conductivity decay during the sample’s hardening was obtained, as shown in Figure 8 for both AC and DC measurements. This behaviour was attributed to a decrement in the ionic conduction mechanism [150], while the hydration reactions consumed the water inside all systems and pore refining during curing.

#### 4.2.3. Flexural Strength

**Figure 9:**
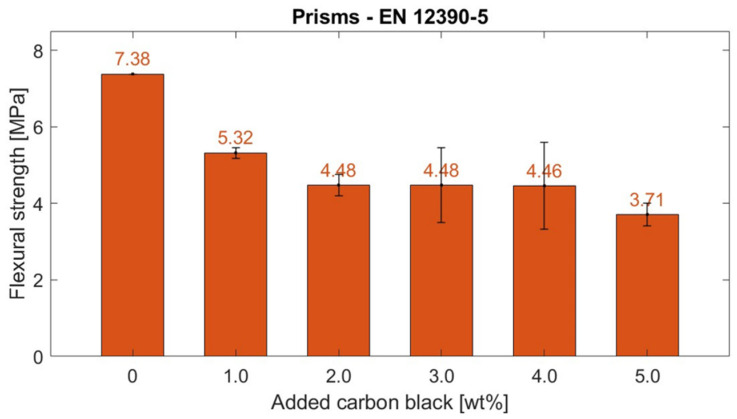
Flexural strength values for CB1, CB2, CB3, CB4, and CB5 CB–cement paste bulk prisms subjected to 3-point bending test. The standard deviation was obtained from testing three identical samples.

Previous works have added carbon-based fillers to improve the mechanical performance of cementitious composites for structural purposes [63]. However, flexural tests conducted in this work presented a lower strength for composites with the CB functional fillers with respect to control specimens, as depicted in Figure 9. At 28 days of age, the CB–cement paste composites showcased a progressive strength reduction from their control value (7.38 MPa). Specifically, when adding 1 wt% CB, the flexural strength of the composite reduced to 5.32 MPa, and the strength plateaued at around ~4.48 MPa for CB2, CB3, and CB4 but reduced to half its control value for CB5 (3.71 MPa). Samples containing higher carbon black concentrations (3 and 4 wt%) exhibited greater standard deviation compared to other specimens, a variation linked to the dispersion challenges of conductive particles. Indeed, some prisms experienced filler agglomeration in certain areas, which resulted in inconsistent mechanical performance [60]. However, this issue was not observed in samples with the highest dosage of carbon black (5 wt%), where the abundant conductive filler uniformly impacted the sample’s properties.

Such a strength decrement can be related to the microscopic behaviour of carbon-based particles, which acted as starting areas for fracture initiation and propagation [49,64,151]. Nonetheless, the designed system was applied as a non-structural coating. Therefore, the flexural strength decrease was not limiting in view of the following monitoring tests.

#### 4.2.4. Adhesion Strength

**Figure 10:**
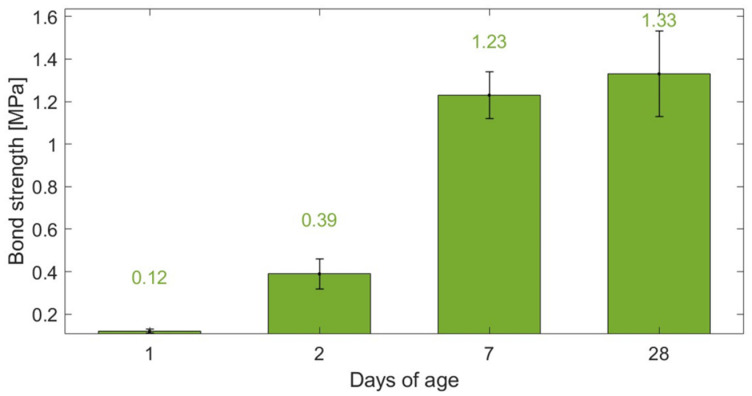
Splitting tensile bond strength between 100 mm × 100 mm × 50 mm concrete substrates and 100 mm × 100 mm × 50 mm CB-based composites. The standard deviation was obtained from testing three identical samples.

Since this study’s innovation lies in using a smart coating to monitor the strain in a concrete substrate, rather than replacing a structural element with a sensing component, the bonding properties between the two composites were analysed. The test involved a cubic 100 mm × 100 mm × 100 mm specimen composed of CB–cement paste and concrete subjected to splitting tensile stress. Figure 10 displays the adhesion strengths of cement paste–CB composites cast onto concrete substrates after three hours. All composites encountered bond mechanism failure at the interface between the two components. This was attributed to the threshold face being the weakest element in this two-part cube. This proved that the tensile strength of the interface was not as strong as the tensile strength of either the coating mix or the substrate mix [124]. In addition, based on the failure mode, it could be concluded that a strong interlocking between the substrate and the coating mix design was absent. This could be attributed to the low roughness of the substrate’s surface, preventing interlocking between the two materials, which also hinders the formation of cement hydration products [152].

Although a longer curing time did not vary the failure mode, as the system cured, the bond strength reached higher values, up to 1.33 MPa at 28 days of age. The adhesion improved from day 1 to day 2—from 0.12 to 0.39 MPa, respectively—with a substantial increment at seven days of age (1.23 MPa). Overall, the bond strength found in this study was within a similar range to other splitting prism applications found in the literature [153]. Enhancements in adhesion might be achieved through increasing the surface roughness of the cured substrate [154,155] and by incorporating fibres into the coating mix design [152,156]. This approach mirrors the fundamental principle observed in the enhanced bond strength between concrete and deformed rebars compared to plain rebars—from 0.98 to 2.2 MPa [157]. As the indentations on deformed rebars create a mechanical interlock with concrete, leading to improved load transfer and reduced slippage [158], introducing textured surface treatments and fibrous materials can significantly elevate the adhesion strength of the CB-based sensing coating to concrete.

### 4.3. Influence of CB Concentration on Composite’s Electrical Properties

**Figure 11:**
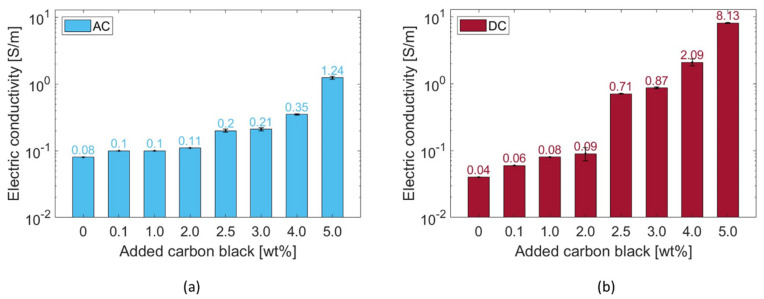
Conductivity values for the CB–cement paste coatings at 28 days of age as a function of CB concentration for (**a**) AC and (**b**) DC. The standard deviation was obtained by testing three identical samples for each mix.

The addition of CB led to a decrease in the electrical resistivity of the specimens. Figure 11 displays how the electrical conductivity of the composites at 28 days of age varied proportionally to the CB filler quantity.

Given the sample’s inner moisture conditions during testing—moisture content = 4% and saturation degree = 90%—the ionic conduction mechanism [18] drove the specific electrical behaviour characterising the pure cement pastes, reaching 8 × 10^−2^ S/m and 4 × 10^−2^ S/m when applying AC and DC, respectively. As the added CB content increased, the electrical properties increased, as well. Nonetheless, in agreement with the percolation theory, such an increment was rather exponential above a 2.5 wt% CB dosage (Figure 11). The conductivity variation between the two electrical application methods was attributed to the progressive stabilisation of the electric field within the system (i.e., polarisation effect) and, so, reduced the voltage drop between the two electrodes, according to Ohm’s law [40]. In this regard, it is worth mentioning that, for future work, biphasic DC could become a practical alternative to limit polarisation [159]. The challenges encountered during the mixing process of 5.0 wt% CB with cement paste did not allow for the investigation of higher carbon black concentrations to achieve a complete percolation curve.

When applying an alternating current, the systems with 0.1, 1.0, and 2.0 wt% added CB had a similar conductivity value of ~0.1 S/m. Similarly, the systems subjected to a direct current witnessed a low increment—with respect to the control—of up to 2.0 wt% added CB (from σ_DC_ = 0.04 S/m to σ_DC_ = 0.09 S/m). Such results proved that cementitious binders with carbon black additions below 2.0 wt% resulted in an insufficient electrical network inside the matrix. However, above 2.0 wt%, the system stabilised its AC conductivity at around 0.2 S/m for both CB dosages of 2.5 and 3.0 wt%. In DC, the system experienced a substantial conductivity increment, i.e., the conductivity at 2.5 wt% was 0.71 S/m, almost eight times the value at 2.0 wt%. Analogously to AC, specimens with 3.0 wt% CB had a similar electrical conductivity (σ_DC_ = 0.87 S/m) to the ones with 2.5 wt%. A subsequent dosage increment to 4.0 wt% CB allowed the composite to reach an electrical behaviour of 0.35 S/m when applying AC and 2.09 S/m for DC measurement. These values described the percolation zone where the highest electrical variation was witnessed (Figure 11), with an experimental percolation threshold (Φ_c_) of around 2.5 wt%. This measurement agreed with filler additions of a similar type, size, and quality found in the literature [36,62]. In addition, such a value was similar to the theoretical threshold defined in the literature for randomly distributed particles [160]. Indeed, by considering 10 nm as the maximum distance between adjacent particles that permits electron hopping, an analytical threshold equal to 10.9 vol% (2.0 wt%) was defined for carbon black particles perfectly distributed throughout the matrix. Nonetheless, as this formulation requires a completely uniform distribution of the conductive particles, such a theoretical value was considered as the lower threshold for the percolation zone. Finally, between 4.0 and 5.0 wt%, the system abruptly reached conductivity peaks of 1.24 and 8.13 S/m for AC and DC measurements, respectively. This high incremental step was in agreement with the percolation theory, i.e., substituting 5.0 wt% of cement with CB led to a stable conductive network throughout the cement matrix—analogous to what was found by Li and Li [69]. This electrical performance was confirmed by Equation (6) [161], which theoretically describes the distance between spherical fillers such as carbon black:

(6) $$d_{s}=\frac{a}{2}{\left(\frac{4\pi }{3\phi }\right)}^{\frac{1}{3}}$$

where *a* is the filler diameter [nm] and *Φ* is the filler concentration [vol%]. The obtained distance varied from a maximum of 177.4 nm for 0.1 wt% of added CB to 52.2 nm for a 5 wt% addition. The calculated interparticle distance at the percolation threshold was 62.9 nm. The percolation curves of Figure 11 agree with Stankovich et al. [162], who defined a power law (Equation (7)) to describe the electrical performance of the composite for filler dosages above the percolation threshold:

(7) $$\sigma =\sigma_{f}{\left(\frac{\phi -\phi_{c}}{1-\phi_{c}}\right)}^{\gamma }$$

where *σ_f_* is the filler’s conductivity [S/m], *Φ_c_* is the percolation threshold [vol%], and *γ* is the universal critical exponent. The conductivity values shown in Figure 11 were fitted with the theoretical distribution described in Equation (7) and the obtained values fell between the AC and DC results, i.e., σ_2.5 wt%_ = 0.24; σ_3.0 wt%_ = 0.43; σ_4.0 wt%_ = 2.35; σ_5.0 wt%_ = 5.41.

**Figure 12:**
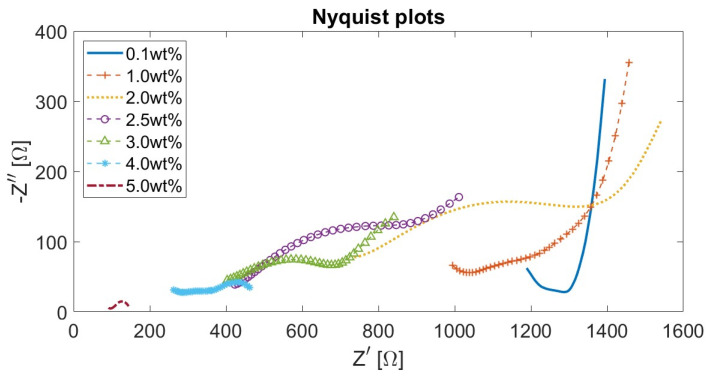
Nyquist plots for all the mixes described in Table 4, at a frequency range of 1 Hz–10^6^ Hz. For CB/cement samples with a carbon black concentration below 4 wt%, all curves are characterised by two semicircles at high frequencies and a straight line at low frequencies. Above such dosage, the semicircle/line shape changes, proving a variation in the conductive mechanism [47].

Additionally, Figure 12 presents the Nyquist plots for CB–cement paste composites with varying CB dosages. As the content of CB filler increased, both the real and imaginary parts of the impedance decreased, reaching low resistance values for CB5. Moreover, since all samples were tested in analogous humidity conditions, all curves were characterised by a straight line at low frequencies that progressively reduced into a semicircle as the CB filler increased. This is attributed to the microscopic conduction mechanism, which is both ionic- and electronic-driven. Thus, proportionally to the conductive phase contribution, the straight line at low frequencies progressively reduces [46]. The presence of the line at low frequencies was likely due to the blocking copper electrode, which led to ions’ (mainly sodium and potassium) accumulation at the interface with the cement paste [163]. Moreover, all curves in Figure 12 shifted to lower resistance values with an increment of CB, proving the values at the cusp points shown in Figure 11. Then, the (electronic) conduction became dominant in CB samples from 4 wt% additions upwards.

Therefore, cement pastes with CB additions of 2, 3, and 4 wt% were investigated as they were part of the percolation curve and better suited to the monitoring purpose of this work [164]. On the other hand, while the addition of 5 wt% CB resulted in a highly conductive system, this may not always translate into high sensing performance as the system can become rather insensitive to electrical changes, rendering it unsuitable for sensing applications [12]. For this reason, coupled with the poorer mechanical properties of the composites with 5 wt% CB, only cement pastes with CB additions of 2, 3, and 4 wt% were considered in the continuation of this study.

### 4.4. Electromechanical Testing

The electrical response of the conductive coatings was evaluated to monitor the strain progress in the elastic region for partially reinforced concrete beams subjected to bending. The stress–strain behaviour confirms the studied elastic domain for concrete beams with rebar reinforcement of up to 5 MPa (3.60 kN).

Within the elastic region, the tested prisms were subjected to 20 cycles—with a loading rate of 50 N/s—between 0.84 MPa, corresponding to 5% of the beam flexural strength and used as preload to ensure the sensing response of the coating, and 3.7 MPa, corresponding to 20% of the sample strength and selected to ensure that the bending occurred in the elastic domain for the composed system. The sensing response of the coatings varied as a function of the CB conductive filler concentration distributed in the matrix as well as the geometry of the composite.

**Figure 13:**
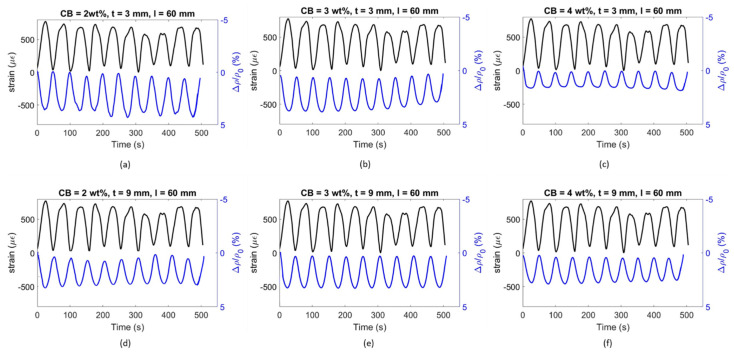
Strain and FCR time histories for (**a**,**d**) CB2, (**b**,**e**) CB3, and (**c**,**f**) CB4 smart coatings applied on partially reinforced concrete beams subjected to cyclic loading. Coating thickness = (**a**–**c**) 3 mm and (**d**–**f**) 9 mm. Gauge length = (**a**–**f**) 60 mm.

Generally, the electric output tended to stabilise as the number of cycles increased because of the polarisation drift [62] and the progressive drying of the water trapped in the system [165]. Han et al. [58] also specified that microdamage could occur in the sample during repeated loading, representing an increment of FCR. As such, only the last 10 cycles were considered when assessing the load sensitivity of the coating in which a stable sensing response was achieved. Figure 13 displays the time progression of strain and FCR when subjected to loading/unloading cycles for 3 and 9 mm thick smart coatings. CB concentrations of 2, 3, and 4 wt% were chosen because of their electrical performance within the percolation zone, as previously defined in Figure 11. The obtained FCR was positive because of the elongation of the conductive matrix under tensile strain that resulted in a resistivity increment with respect to the initial value and, thus, resulted in a positive FCR—according to Equation (5) [12].

**Figure 14:**
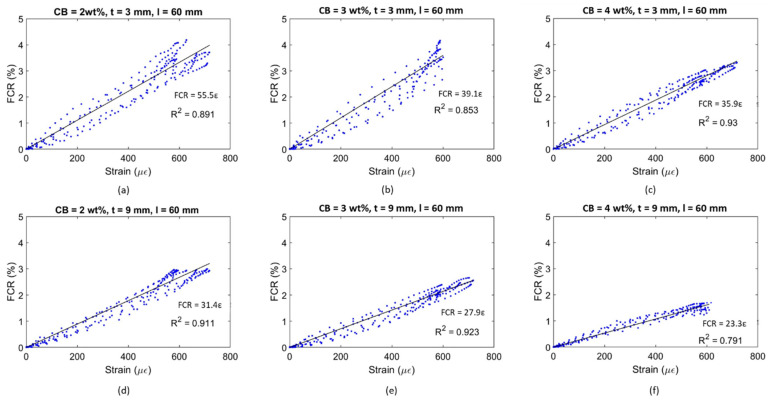
FCR vs. strain for substrates/coatings subjected to a monotonic flexural load (last 10 loading cycles), and linear fit models, as a function of varying CB dosage: (**a**,**d**) 2 wt%; (**b**,**e**) 3 wt%; and (**c**,**f**) 4 wt%. Coating thickness = (**a**–**c**) 3 mm and (**d**–**f**) 9 mm. Gauge length = (**a**–**f**) 60 mm. Gauge factor was obtained by fitting the FCR–strain relationship for three samples of each configuration.

The FCR amplitude for each strain cycle can be related to the coating’s strain sensitivity. The graphs in Figure 14 plot the electrical response of the smart coatings against the strain development for the bottom layer of the concrete substrate.

The general trend saw a progressive sensitivity reduction when increasing the filler concentration. Indeed, smart coatings with 2 wt% CB and 3 mm thickness showcased a gauge factor of 55.5, which was 30 and 35% higher than the values obtained for CB3 and CB4, respectively. A similar response was also observed for 9 mm thick coatings, where the sensing response of CB2, 31.4, was greater than the strain sensitivity achieved by 3 and 4 wt% CB, i.e., 11 and 25% higher, respectively. Considering the percolation curve (Figure 11), this performance was in agreement with the literature; it has been reported that filler concentrations closer to the lower region of the percolation zone could lead to greater strain sensitivity compared with higher thresholds within the same percolation zone [164]. For example, in this study, 2 wt% CB provided the highest gauge factor when compared to 3 and 4 wt% even though such concentrations of CB better enhanced the electrical conductivity. To elaborate, when adding higher concentrations of CB conductive filler (e.g., 4 wt%), the number of conductive pathways increases [166], and, therefore, the system becomes so conductive that it develops lower strain sensitivity to applied loads [12]. Therefore, the addition of CB at 2 and 3 wt% led to a partially connected—yet conductive enough—path for the electrons to travel within the cementitious composite [58]. Finally, cement coatings with 3 mm thickness resulted in the most responsive composite when compared to 9 mm coatings. The sensing variation between the two coating thicknesses for CB2, CB3, and CB4 was 43, 29, and 35%, respectively. This variation could be attributed to the fact that the electrical response of the sensing coatings was correlated with the maximum flexural strain experienced by the concrete substrate rather than the strain in the coatings. Based on existing literature, it could be posited that lower strain propagation occurred in the thicker coatings and higher strain propagation in the thinner coatings, thus resulting in a different FCR and GF in each case [102,167]. For example, a higher strain sensitivity was reported by Baeza et al. [102] for carbon-fibre-based coatings with 0.5 mm depth compared to 2 mm thick ones (i.e., gauge factors of 178.9 and 64.8, respectively) when attached to the tensile section of an RC beam subjected to bending due to higher strain transmission.

**Figure 15:**
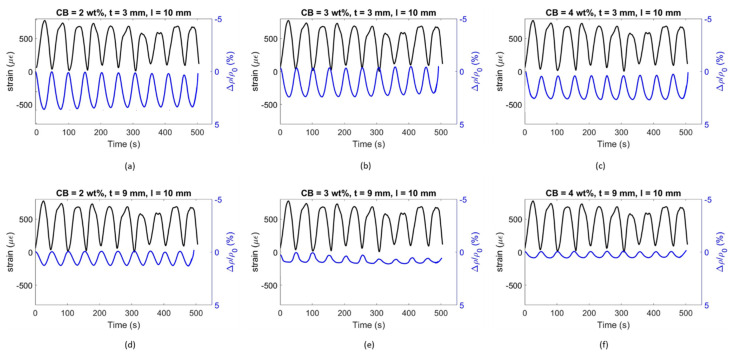
Strain and FCR time histories for (**a**,**d**) CB2, (**b**,**e**) CB3, and (**c**,**f**) CB4 smart coatings, applied on partially reinforced concrete beams subjected to cyclic loading. Coating thickness = (**a**–**c**) 3 mm and (**d**–**f**) 9 mm. Gauge length = (**a**–**f**) 10 mm.

**Figure 16:**
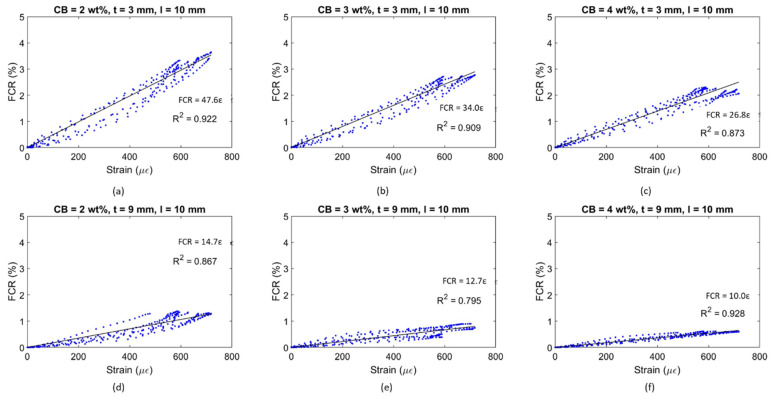
FCR vs. strain for substrates/coatings subjected to monotonic flexural load (last 10 loading cycles), and linear fit models, as a function of varying CB dosage: (**a**,**d**) 2 wt%; (**b**,**e**) 3 wt%; and (**c**,**f**) 4 wt%. Coating thickness = (**a**–**c**) 3 mm and (**d**–**f**) 9 mm. Gauge length = (**a**–**f**) 10 mm. Gauge factor was obtained by fitting the FCR–strain relationship for three samples of each configuration.

An alternative coating type was tested to properly assess the influence of the setup on the flexural strain sensitivity property. By placing electrodes at a 10 mm distance, the coatings were investigated in relation to their sensing capability in a shorter constant bending region. Similar to Figure 13 and Figure 14, Figure 15 and Figure 16 present the time histories of FCR and strain as well as their relationship for this new set of smart coatings. The obtained FCR amplitudes for this investigation were lower than the previous configuration but showed an analogous pattern with a descending sensitivity when increasing the CB dosage and the coating thickness. For 3 mm coatings, the addition of 2% CB led to a gauge factor of 47.6, which was higher compared to CB3 and CB4 (34.0 and 26.8, respectively). These results were 14, 13, and 25 times smaller, respectively, when compared to coatings with a greater gauge length. Similar to the results portrayed in Figure 14, increasing the coating thickness to 9 mm resulted in poorer sensitivity, i.e., 14.7, 12.7, and 10.0 for CB2, CB3, and CB4, respectively. These values were approximately 55% lower than the same coatings with longer gauge lengths. The reduction observed in strain sensing performance by shortening the gauge length could be attributed to the strain and current density distribution inside the sensing coating. When placing electrodes at a 10 mm distance, the sensing response was limited to the strain experienced by the coating in this short section. In contrast, testing the electrical response through electrodes spaced at 60 mm enabled a larger area to be monitored in which the coatings experienced larger strain. Furthermore, the gauge length has been correlated with the current penetration in the binder, which impacts conductivity measurements [168] and, in turn, strain-sensing performance [129,169]. In addition, placing the electrodes near the edges of the loading area (i.e., 60 mm in the four-point bending test) could lead to higher contact resistance [169] and, therefore, to a greater increase in FCR.

**Figure 17:**
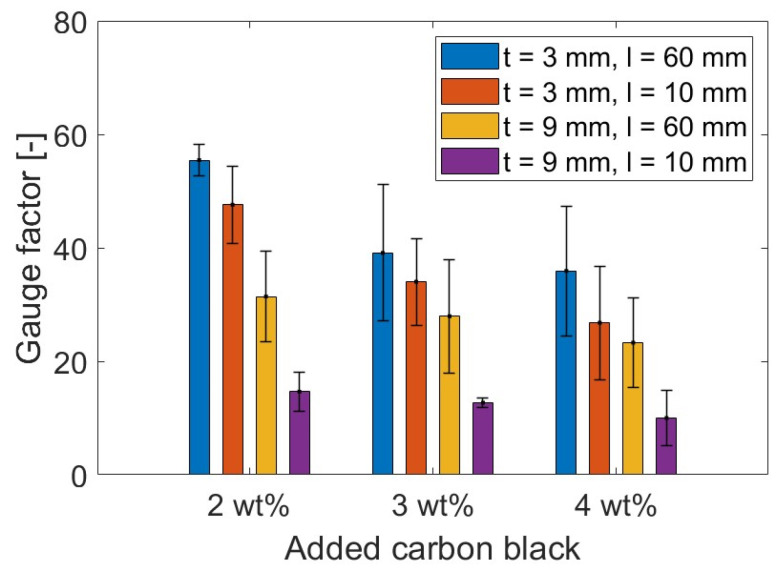
The gauge factor for 2, 3, and 4% CB dosages in the cement-based coating for varying CB concentrations, coating thicknesses, and electrode spacing scenarios. The standard deviation was defined by comparing gauge factors of samples with the same geometrical setup and filler concentration. Refer to online version for colour representation.

It could be observed that some of the plots in Figure 14 and Figure 16 were characterised by a non-linear behaviour at the higher end of the loading range. Although the tests were conducted in the elastic zone, some samples might have been affected by a weaker performance, therefore entering the plastic domain for strains above 600 με. All electromechanical results obtained in this study are shown in Figure 17. The standard deviations of sensing performance in smart coatings increase with the carbon black dosage, with notable variability in CB4 specimens. This variability is linked to the effectiveness of carbon black dispersion, which may have led to agglomerations in certain sections of the coating, disrupting the electronic pathways in the conductive network. Thus, analogous to the flexural strength behaviour (Figure 9), the electronic influence of carbon-based additives is closely related to their distribution within the cementitious matrix [83].

Figure 17 shows a decreasing strain sensitivity for coatings with higher carbon black concentrations. Similarly, Li and Li [69] investigated mortars with CB additions of 2.5, 5, and 10 vol% in tension and obtained the following gauge factors: 52 ± 19 for 2.5 vol%; 247 ± 24 for 5.0 vol%; and 105 ± 12 for 10 vol%. This descending trend was attributed to the variation in tunnelling effects and percolation phenomena inside the matrix for increasing carbon black concentrations [166]. In fact, higher dosages of conductive fillers led to a denser network that became less responsive to any load variation [12]. The high sensitivity values obtained by Li and Li [69] were attributed to the presence of fly ash and silica fumes, which increased the strain-sensing performance by creating a denser matrix [60]. It should be pointed out that the sensing response was examined under pure tension whereas this study investigated the electrical response of coatings under bending, where factors such as the electrode configuration, which are known to influence the sensing performance, become more prominent [14]. If compared to bulk applications under flexural loading, the stress sensitivity achieved in this study ranged between 0.25 and 1%/MPa, which was within the same order of magnitude as that found by Dong et al. [83] for mortars with 0.5 wt% added CB, 0.06–0.55%/MPa, and by Guo et al. [92] for a filler content of 1.5 wt% (1.8%/MPa). Therefore, by demonstrating comparable sensing capabilities to bulk applications, the CB-based systems developed in this study proved to be suitable in the field of strain sensing.

With respect to previous works on coatings for flexural strain sensing, the smart systems investigated in this work displayed similar trends and were characterised by comparable changes in electrical properties. The gauge factor attained for 3 mm coatings in this paper was 55.5, which is within a reasonable range of the GF reported by Baeza et al. [102] (i.e., 64.8) for coatings with a 2 mm thickness. Since the gauge factor from other studies involving sensing coatings is not available, the FCR can be used to compare experimental findings within similar strain and loading regions. Nonetheless, it should be noted that definitive conclusions cannot be drawn from comparing the maximum FCR amplitude alone as the GF should be the primary method of evaluating strain sensors. The FCR obtained in this investigation for all sensor designs ranged between 1% and 4% for an applied strain of 600 με and a load of 4 MPa. Kim et al. [104] reported a maximum sensing response of 2% for 10 mm thick MWCNT-based coatings within a stress range of 30% of flexural strength. Durairaj et al. [103] obtained FCR values of between 10% and 15% for a strain range similar to the one investigated in this study by employing a 6 mm deep mortar layer with various combinations of brass and carbon fibre. Wen and Chung [101] obtained a maximum FCR amplitude of 0.15% on the tension side for a 5 mm thick coating with 0.5 wt% added carbon fibre for a beam deflection of about 0.09 mm. The sensing response also agreed with the values defined by Qiu et al. [105] (i.e., 2.5%) for CNT-CB mortar coatings on the tension side of elastic bending. Thus, the FCR values in this study are within a broad range of values found in the literature.

Moreover, similar to other sensing techniques such as sensing sheets [167] and strain gauges [170], it is expected that the response of the sensing coatings can be influenced by the interfacial bonding and degree of strain transfer with the substrate. Specifically, a mismatch in the stiffness and Poisson’s ratio of the coating and the substrate can lead to low material compatibility between the two. In this study, as the sensing coating is cement paste, while not directly tested, it can be assumed that its elastic modulus [166] would be lower than that of the concrete substrate [114,171]. Based on the existing literature [172,173,174,175], having a coating with a lower elastic modulus than the substrate could result in cracking and delamination of the coating. To counter these issues, we would need to ensure an adequate bond between the two materials and a sufficient coating thickness to resist cracking. In this work, the bond strength was measured as 1.33 MPa, which is regarded as a serviceable adhesion strength according to BS EN 1504-3 [176] (ASTM C1583/1583M [177]) [178]. Furthermore, neither 3 mm nor 9 mm coatings displayed signs of cracking or delamination during curing and testing, verifying that the chosen thicknesses were able to withstand cracking and delamination [179]. While the mismatch in properties did not play a significant role in this study, this may become a more relevant issue in applications where greater loads are applied and when the coatings are exposed to harsher environments [180]. Future work could involve improving our sensor’s design to minimise the adverse effect of mismatch in properties by increasing the bond strength of the coating; this will be achieved by adding fine aggregates and fibres to the mix design and investigating different surface preparation techniques. However, it should be noted that—in practice—a mismatch between overlays and parenting substrates will be difficult to avoid due to the wide range of structures in the built environment as well as the fresh properties and geometry of coatings, etc. [106,172,181,182]. Proper curing, even in field applications, should be ensured to prevent excessive shrinkage and cracking, and to guarantee the coatings’ proper integration with the substrate. Deploying a sensor network can allow for effectively monitoring large areas and identifying potential coating failures or malfunctions caused by bonding issues. Overall, constant research is being carried out in the field of concrete overlays and repairs to investigate the impact of these phenomena. We, therefore, acknowledge this is a known limitation in the field of cementitious sensing coatings and one that should be accounted for in the developmental and deployment stages. Grades and different types of sensing coatings could be developed depending on the intended applications, to ensure proper compatibility.

In summary, this study has broadened the current research into self-sensing materials through the development of carbon black cementitious sensing coatings. The fabrication process here presented followed a simple mixing procedure and the application method was explained in detail. This approach can serve as a sustainable and cost-effective alternative to the use of conventional bulk carbon-based self-sensing materials while at the same time achieving a reliable monitoring performance. Specifically, this study has investigated the influence of filler concentration, thickness, and electrode spacing on the strain sensitivity of smart coatings. The results provided the optimal configuration to be employed to monitor the elastic behaviour of a concrete substrate subjected to a flexural load. It is important to point out that the coatings were examined under controlled moisture and temperature conditions. Future studies shall focus on stabilising the monitoring performance of smart coatings with respect to field conditions such as by impermeabilising the coatings with epoxy and compensating measurements with the use of reference sensors. Additionally, given the microstructural complexity of the electrical network under a flexural load, further micromodelling analysis is required to numerically prove the tunnelling conduction behaviour when the entire system is subjected to bending.

## 5. Overview and Recommendations

This study has investigated the influence of CB as a functional filler on cement paste composites with respect to their electrical properties. The strain-sensing capability was assessed by employing the CB/cement composite as a conductive coating onto concrete substrates. The most suitable coating configuration was defined in relation to the highest strain-sensing response for concrete substrates in bending. Additionally, to better understand the mechanical performance of CB-based cementitious systems, this work focused on assessing the viscosity, hydration development, microstructure, and flexural and adhesion strength of these smart coatings. Dosages of CB of 2, 3, and 4% by weight of cement were used based on the percolation theory.

The findings reveal a significant influence of carbon black on cement paste composites at the physical level. For instance, the incorporation of CB resulted in proportional increases in plastic viscosity across the concentrations tested, highlighting the poor hydrophilic properties of the filler. From the cement paste viscosity value of 255.1 mPa∙s, this increased to 484.6 mPa∙s for CB2, 523.8 mPa∙s for CB3, and 671.2 mPa∙s for CB4. Thus, the production of CB/cement smart coatings required a certain amount of superplasticiser to achieve sufficient workability. The addition of superplasticiser at 10% by weight of CB led to rheological properties closer to the control value for CB2 and CB3 (i.e., 261.6 and 292.1 mPa∙s, respectively). In contrast, the use of dispersant for mixes with a higher filler concentration did not greatly facilitate the overall workability due to the conductive particle’s effect on free water.

The early hydration reactions benefitted from the presence of CB particles serving as nucleation sites and thereby accelerating the hydration process. This acceleration was evidenced by a 0.5 h shift in the peak heat of hydration with respect to the control. Electrical conductivity measurements, both in AC and DC, provided insight into the hydration growth of the cementitious samples over a 28-day period. Despite an initial exponential decay in conductivity, a plateau was reached, underscoring the consistent reduction in conductivity across all CB dosages due to water consumption within the system.

The addition of carbon black also had a significant impact on the flexural strength of the composite. Compared to the cement paste strength of 7.38 MPa, samples with 1.0 wt% CB had a lower flexural strength of 5.32 MPa. For higher dosages of 2.0, 3.0, and 4.0 wt%, the strength was further weakened to ~4.48 MPa. The cementitious composite with 5.0 wt% CB reduced to half the strength of the control (i.e., 3.71 MPa). This behaviour was related to the presence of weaker CB particles, which acted as starting zones for fracture initiation and propagation.

For what concerns the electrical performance of the composite binder, the addition of CB in cement pastes increased the electrical conductivity for both AC and DC measurements. While CB0.1, CB1, and CB2 did not experience a substantial electrical enhancement compared to cement paste (σ_AC_ = 0.08 and σ_DC_ = 0.04 S/m), the electrical conductivities of samples with an added 2 wt% were 2.5 and ~18 times higher than the control for AC and DC, respectively. The electrical conductivity steadily increased up to 3 wt% of CB filler content (σ_AC_ = 0.21 S/m and σ_DC_ = 0.87 S/m) and peaked at 5 wt% of CB (σ_AC_ = 1.24 S/m and σ_DC_ = 8.13 S/m), leading to a stable network throughout the hybrid system. This behaviour was attributed to the reduced bulk resistance, which was observed from the semicircle in the Nyquist plot during the electrochemical impedance analysis.

This study’s investigation into the strain-sensing capabilities of CB/cement composites yielded a gauge factor reduction for increasing filler dosages within the percolation zone. In agreement with the literature, adding 2 wt% of CB—lower threshold of percolation zone—resulted in higher sensitivity than composites with 4 wt% of CB—higher threshold of percolation zone. For 3 mm thick coatings and electrodes distanced at 60 mm, the obtained gauge factor progressively reduced from 55.5 ± 2.7 for CB2 to 39.1 ± 11.8 for CB3, reaching a weaker response of 35.9 ± 11.4 by CB4. The same pattern was defined for thicker coatings, i.e., 31.4 ± 8.0, 27.9 ± 10.1, and 23.3 ± 7.8 for CB2, CB3, and CB4, respectively. Similarly, an electrode distancing of 10 mm led to higher strain sensitivity for coatings with 2 wt% of carbon black (47.6 ± 6.8 and 14.7 ± 3.5 for 3 and 9 mm thick coatings, respectively) compared to the response achieved by samples with carbon black additions of 3 and 4 wt% for both 3 (GF_3 wt%_ = 34.0 ± 7.7 and GF_4 wt%_ = 26.8 ± 10.0) and 9 mm thick systems (GF_3 wt%_ = 12.7 ± 0.9 and GF_4 wt%_ = 10.0 ± 4.9). When comparing the strain-sensing responses of the different coating configurations tested for smart coatings, a better electrical–physical relationship was achieved when placing the electrodes along the entire constant bending moment region (i.e., 60 mm) and employing a 3 mm thick coating, reaching a maximum gauge factor of 55.5 ± 2.7 for CB2. In contrast, when choosing CB dosages in the higher end of the percolation zone (4 wt%), using a smaller distance between the electrodes (i.e., 10 mm) and applying a thicker coating of 9 mm led to the weakest sensing response of 10.0 ± 4.9.

In summary, this work found that, among various configurations, 3 mm thick smart coatings with 2 wt% of added carbon black and an electrode distancing along the constant bending region gave the highest strain-sensing response when applied to a 40 mm × 40 mm × 160 mm concrete beam in bending. This configuration offers promising potential for monitoring civil engineering structures, providing high sensitivity and good interaction with cementitious substrates.

This study serves as a foundation for the production and application of smart coatings in concrete monitoring. Future work should focus on ensuring the impermeability of the smart coating response to external working conditions, e.g., moisture and temperature, to achieve a greater degree of repeatability and reliability of the sensing response in different environments. The sensing performance developed in this research was dependent on the presence of water inside the smart coating. Therefore, the water influence on the sensing response needs to be stabilised with respect to changing environmental conditions by means of a waterproof layer. Further steps in this direction involve numerically investigating the impact of the mismatch in the Young’s modulus and Poisson’s ratio between the coating and the substrate. The work presented in this paper primarily focused on the elastic domain and, therefore, additional research is essential to investigate the damage-sensing performance of these coatings for cementitious substrates in higher loading conditions. Moreover, future studies could involve the application of such a hybrid material in combination with self-healing systems, thus assessing the healing performances under various loading conditions. Additionally, smart coatings could be employed as a non-destructive technique for monitoring corrosion in reinforced concrete by measuring the changes in electrical resistivity in the presence of sodium chloride—for non-waterproofed coatings—or by relating the electric response to the rebar corrosion within the reinforced concrete substrate.

## 6. Conclusions

This study has explored the use of conductive carbon black (CB) as a functional filler in cement paste composites, focusing on its strain-sensing capability when employed as an external coating. Key findings include the following:

- The incorporation of CB into cement pastes influences workability and mechanical strength adversely, while it beneficially accelerates the hydration process.
- Low dosages of carbon black (i.e., 2.5 wt% or 13.5 vol%) in cement pastes are effective in increasing electrical conductivity.
- Carbon black-based smart coatings demonstrated a significant strain monitoring capability for concrete structures, highlighting their applicability in assessing and ensuring the structural integrity of concrete elements.
- The optimal trade-off between physical, electrical, and electromechanical properties was accomplished by smart cementitious sensors including 2 wt% of carbon black.
- A thinner sensor configuration (thickness = 3 mm) with electrodes distanced along the entire constant bending moment region (gauge length = 60 mm) provided the highest flexural strain sensitivity.

In summary, this study underscores the potential of carbon black-based smart coatings in the field of structural health monitoring. The practical implications of this research extend significantly within the construction industry, particularly in enhancing the durability and safety of infrastructure. Carbon black-based smart coatings offer a practical solution for early detection of structural weaknesses, potentially reducing maintenance costs and extending the lifespan of concrete structures. This novel approach was characterised by an enhanced monitoring sensitivity with respect to existing technologies and solid adhesion with the concrete substrate. Potentially, the integration of these coatings with existing structural health monitoring systems could help in accomplishing more intelligent, responsive infrastructure capable of adapting to changes in loading conditions. Further development is required to improve the coatings’ reliability and repeatability in various environments and to explore their integration with self-healing materials and corrosion monitoring techniques. The evolution of smart coatings into systems that not only monitor but also respond to detected damage represents a critical approach to sustainable construction practices, aiming to fill the industry’s need for smart/adaptive materials, in view of safer and more resilient infrastructure.

## Figures and Tables

**Table 2:** Carbon black properties as per the manufacturer.

Appearance (colour)	Black
Form	Powder
Ash (%)	≤0.50
Electrical resistivity (Ω∙cm)	≤0.25
pH	7.6
Moisture (%)	0.12
Average particle size (nm)	42
Surface area (m^2^/g)	75
Bulk density (g/L)	170–230

**Table 3:** Mix design of the concrete substrate tested in this study (kg/m^3^).

Cement	Water	Fine Aggregate	Coarse Aggregate
395	178	829	1184

**Table 4:** Mix design of the costing composition tested in this study (kg/m^3^). Annotation: CTRL = control sample; CB2—CB = carbon black, 2 = percentage by weight of cement.

Name	Cement	Water	Carbon Black	Dispersant	CB Dosage [wt%]	CB Dosage [vol%]
CTRL	2950.0	1327.5	0	0	0	0
CB0.1	2948.6		2.9	0.3	0.1	0.6
CB1	2935.6		29.2	2.9	1.0	5.9
CB2	2921.2		57.8	5.8	2.0	11.1
CB2.5	2914.0		72.0	7.2	2.5	13.5
CB3	2906.8		85.9	8.6	3.0	15.7
CB4	2892.4		113.5	11.3	4.0	19.9
CB5	2878.0		140.5	14.1	5.0	23.6

## Data Availability

The data that support the findings of this study are available from the authors upon request.
